# Relinquishing Owners Underestimate Their Dog's Behavioral Problems: Deception or Lack of Knowledge?

**DOI:** 10.3389/fvets.2021.734973

**Published:** 2021-09-10

**Authors:** Lauren Powell, Deborah L. Duffy, Katherine A. Kruger, Brittany Watson, James A. Serpell

**Affiliations:** ^1^School of Veterinary Medicine, University of Pennsylvania, Philadelphia, PA, United States; ^2^Office of Institutional Research, University of the Arts, Philadelphia, PA, United States; ^3^Office of the University Secretary, University of Pennsylvania, Philadelphia, PA, United States

**Keywords:** dog, relinquishment, animal shelter, human-dog bond, C-BARQ

## Abstract

Undesirable behavior is a leading cause of canine relinquishment. Relinquishing owners could provide valuable information about their dog's behavior, although the reliability of their reports has been questioned by the sheltering community. This study aimed to investigate (a) whether relinquishing owners' reports of dog behavior differed based on the behavioral screening method; (b) whether relinquishing owners' reports were impacted by the confidentiality of their responses; and (c) whether relinquishing and non-relinquishing owners perceived the behavior of their dogs differently. The sample included 427 relinquished dogs from three animal shelters and 427 pet dogs, matched for sex and breed. Owners responded to a direct question about whether they were experiencing problems with their dog's behavior and completed the mini C-BARQ which includes 42 questions about the frequency and severity of specific canine behaviors in various circumstances. More than two-thirds (69.3%) of relinquishing owners indicated they were not experiencing problems with their dog's behavior when asked directly, compared with only 34.5% of pet owners. Yet, relinquished dogs had significantly higher (less desirable) scores than pet dogs across most C-BARQ scales. The disparity between C-BARQ scores and the single, direct question does not appear to be the result of deliberately biased reporting by relinquishing owners as the perceived confidentiality (or lack thereof) did not affect their responses (*X*^2^ = 1.44, *p* = 0.97). It is possible that relinquishing owners had less understanding of dog behavior and did not recognize behavior problems as a problem. Our findings support the use of standardized behavioral questionnaires, such as the mini C-BARQ, to collect behavioral information from owners at the time of relinquishment and highlight opportunities for animal shelters to reduce relinquishment by assisting owners to recognize and manage behavioral problems.

## Introduction

Undesirable behavior is prevalent among pet dogs, with 72–85% of dogs estimated to exhibit at least one type of problem behavior (1, 2). These behaviors may reflect normal canine behavior that owners simply find undesirable, such as barking, or they may arise from medical or behavioral pathology, such as generalized anxiety disorder or compulsive behavior (3). Canine behavior problems can often be indicative of a poor welfare state (4, 5) and lead to an increased risk of euthanasia. Undesirable behavior is the leading cause of mortality among privately owned dogs under the age of 3 years (6, 7). Problem behaviors have also been associated with reduced owner attachment and satisfaction (8–11) which may threaten the human-animal bond and place the dog at increased risk of relinquishment as behavioral issues are a leading cause of canine relinquishment worldwide (12–14). Many unsuccessful animal adoptions, in which animals are returned to the shelter post-adoption, can also be attributed to behavioral issues (15–17).

Animal shelters often assess the behavior of incoming dogs to help determine their suitability for adoption and match them with adoptive owners based on their behavioral needs and the adopter's ownership preferences (18). Concerns have been raised about the use of standardized behavioral assessments in shelters as some evidence suggests they have a poor ability to predict the future behavior of dogs in the home environment (19). Other researchers argue that standardized assessments provide useful behavioral information, particularly when combined with reports from previous owners (18). Survey instruments, such as the Canine Behavioral Assessment and Research Questionnaire (C-BARQ), have been used successfully to collect behavioral data from relinquishing owners (20, 21). Significant correlations have been documented between C-BARQ scores and canine behavior captured during shelter behavior assessments (22, 23). The shortened or “mini” version of the C-BARQ (42-items compared with the 100-item C-BARQ) has also been shown to discriminate between shelter dogs based on their outcomes (adopted/euthanized), and mirrors the assessment of shelter staff regarding the presence (or absence) of aggression, i.e., dogs that showed aggression in the shelter also had high scores on the aggression C-BARQ subscales as reported by the owner (20).

Despite these findings, widespread doubts exist within the animal sheltering community regarding the value and reliability of behavioral information provided by relinquishing owners (21, 24, 25). Such doubts have been partly reinforced by the conflicting results of previous research (20, 21). An initial study of 54 relinquishing dog owners found that owners who believed the information they provided was confidential (i.e., not shared with the shelter) reported higher rates of owner-directed aggression and stranger-directed fear on the C-BARQ than owners who were informed that the information would be shared with the shelter (21). The latter group may have misrepresented their dog's behavior, either due to social desirability biases or in an attempt to conceal the presence of behavior problems that might contribute to the pet's euthanasia. However, a second, larger study including 443 relinquishing owners failed to replicate these initial findings. Instead, confidential and nonconfidential groups of relinquishing owners had similar scores across all C-BARQ subscales (20). The authors of this study speculated that the difference in results may have arisen from the way in which the C-BARQ was administered (face-to-face interview in the preliminary study compared with self-completion in the latter). As relinquishing owners' reports appear to differ depending on the manner in which behavioral data is collected, we hypothesized that the quality of owner reports may also depend on how the topic of behavior problems is addressed at the time of pet relinquishment. For example, the C-BARQ, which was designed to reduce bias by asking owners to report the frequency or severity of behaviors in specific situations and contexts in the recent past (26), may evoke different responses from relinquishing owners compared with a direct question about the dog's behavior problems.

To test this hypothesis, the current paper utilizes previously unpublished data from the sample of relinquishing owners in the second of these studies (20) to compare owner's self-completed mini C-BARQ responses with their responses to a single direct written question regarding their dog's behavior problems. We also explore whether both sets of responses (mini C-BARQ and direct, written behavioral question) were influenced by the owner's perception of the confidentiality of the information they provided. The information provided by relinquishing owners may also be affected by their understanding of dog behavior as previous studies have found significant differences between owners in their perceptions of dog behavior (27–29). To characterize relinquishing owners' perceptions of dog behavior and elucidate possible differences between relinquishing owners and current dog owners, we compared the responses of the former with a matched sample of dog owners gleaned from the C-BARQ database at the University of Pennsylvania.

## Materials and Methods

### C-BARQ

This study forms part of a larger study that was designed to evaluate the mini C-BARQ as a tool for screening the behavior of relinquished dogs (20). The mini C-BARQ was developed using step-wise removal to reduce the number of questions in the standard 100-item C-BARQ, whilst ensuring the shortened version maintained adequate internal reliability across the 14 established C-BARQ subscales (20). Through this process, the standard C-BARQ was shortened to a 42-item version which encompasses the 14 standard subscales and 9 miscellaneous items (described in detail in the Supplementary Table 1) which can be completed in <10 min (20). Relinquishing owners were asked to report the frequency or severity of specific behaviors in certain circumstances in the recent past. Each question could be answered on a 5-point scale from 0 (never/no signs) to 4 (always/extreme signs). If owners had not observed their dog in the described situation, they were instructed to leave the item blank. To calculate subscale scores, item scores within each subscale were summed and averaged. Two questions in the training difficulty subscale were reverse scored so that higher scores reflected less desirable behavior across all subscales and miscellaneous items. Mini C-BARQ scores are highly correlated with those of the standard, 100-item C-BARQ (20) which has been used extensively in the literature (26, 30–33).

Prior to completing the mini C-BARQ, owners were also asked to provide their dog's age, breed, sex, neuter status, and source of acquisition, and to indicate whether they were currently experiencing any problems with the dog's behavior. The response options for this question were: “No problems,” “Only minor problems,” “Moderate problems,” and “Serious problems.” In several cases, relinquishing owners did not answer the questions regarding their dog's age (*n* = 4) or breed (*n* = 12), so we used the age/breed that was provided by the participating shelter. The number of missing cases for each subscale and miscellaneous item are provided in Supplementary Table 2.

### Relinquishing Owners

Three animal shelters participated in the study over a 12-month period. Each shelter was located in a different region of the United States, including a private, urban shelter in California (*n* = 198), a rural, municipal shelter in Colorado (*n* = 85), and a private shelter in Missouri (*n* = 160). Shelter staff invited owners to participate voluntarily in the study at the time of relinquishment. To be eligible to participate, owners must have owned the dog for at least 2 months prior to relinquishment, not be relinquishing the dog specifically for euthanasia, and understand sufficient English to complete the survey without the help of shelter staff.

The mini C-BARQ was distributed to relinquishing owners with three different introductory preambles presented in bold font and in a random order. The first preamble stated that participants' responses, “may be used by shelter staff when evaluating your dog for possible adoption.” Owners who received this preamble formed the non-confidential group. The second preamble stated that owners' responses, “will NOT be viewed by employees of the shelter, and are completely anonymous” (confidential), and the third preamble did not specify how or if the information might be used or shared (not specified). For the non-confidential group, a debriefing statement was included on a separate page at the end of the C-BARQ to inform participants that their responses were in fact confidential, and that the introductory preamble was untrue. To ensure participants did not read the debriefing statement prior to completing the C-BARQ, a message was displayed at the top of the page asking participants to make sure they had answered all questions before proceeding. Once completed, participants were asked to seal their responses in postage-paid envelopes addressed to the University of Pennsylvania and to hand them to the staff person who assisted them.

### Case-Control Matching

A sample of control dogs were selected from the online C-BARQ database to match relinquished cases at a 1:1 ratio. Pet owners can voluntarily sign up and complete the C-BARQ online (https://vetapps.vet.upenn.edu/cbarq/), and their responses are then stored in the online C-BARQ database. Control dogs, henceforth referred to as pet dogs, must have been owned for at least 2 months at the time of C-BARQ completion. They were then matched for primary breed, sex, source of acquisition (where possible) and neuter status (where possible). At a minimum, the dogs were matched for breed and sex. Where there were multiple possible control dogs in the database, a pet dog was randomly selected in Microsoft Excel using a random number generator. If the relinquished dog's breed was listed as a mix, cases were matched based on the primary breed. For example, “Labrador mix” from the relinquished database was matched with “Labrador Retriever” from the pet owner database. The only exception was “Pit Bull mix” which was matched directly as this category was also listed in the pet owner database. If the dog's breed was not designated, e.g., “mixed,” it was matched with “mixed breed.” The list of dog breeds is provided in Supplementary Table 3.

To compare the C-BARQ scores of the relinquished dogs with the matched sample of pet dogs, we extracted pet dog owners' responses to the relevant questions from the 100-item C-BARQ. We then calculated subscale scores according to the 42-item, mini C-BARQ system meaning we reverse scored two questions in the trainability scale to produce a training difficulty scale in which higher scores were reflective of poorer trainability. In some cases, the mini C-BARQ includes a single question that corresponds to two questions in the standard, 100-item C-BARQ. For example, the mini C-BARQ asks respondents to indicate their dog's level of fearfulness “when approached directly by an unfamiliar dog,” whereas the standard C-BARQ includes two questions regarding the dog's fearfulness “when approached directly by an unfamiliar dog of the same or larger size” and “when approached directly by an unfamiliar dog of a smaller size.” In these situations, we summed and averaged the two responses from the standard C-BARQ. Like the relinquishing owners, pet dog owners were also asked a direct question about whether they were experiencing any problems with their dog's behavior prior to completing the C-BARQ.

### Statistical Analyses

Length of ownership was calculated as the number of months between the dog's age at acquisition and evaluation. Data were missing for 79 relinquishing owners who did not provide their dog's acquisition age or evaluation age. Descriptive statistics were calculated, and the data were assessed for normality using visual assessment of histograms. Pearson's chi-squared tests were used to compare sex, neuter status, source of acquisition, age at evaluation, and owners' perceptions of behavior between relinquished and pet dogs. Relinquishing owners' perceptions of dog behavior were also analyzed based on the perceived confidentiality of their reports and the shelter at which they relinquished their dog using Pearson's chi-squared tests. *Post-hoc* analyses using standardized residuals were performed following a significant Pearson's chi-squared test. Length of ownership was assessed using a Mann–Whitney *U*-test. Where data were normally distributed, independent sample *t*-tests were used to compare differences in C-BARQ subscales between relinquished and pet dogs, including excitability, training difficulty, chasing and energy level. Mann–Whitney *U*-tests were used to compare all other C-BARQ subscales and C-BARQ miscellaneous items. Variables that were significantly different between relinquished dogs and pet dogs in the univariate analyses were included in binary logistic regression models to investigate C-BARQ subscale scores and miscellaneous item including age, neuter status, and length of ownership. Sex was also included. Statistical analyses were conducted in IBM SPSS Statistics (IBM SPSS Statistics for Windows, version 24). *P* < 0.05 were considered statistically significant.

## Results

Four-hundred-forty-three relinquishing owners completed the mini C-BARQ, although 16 owners were excluded as they had a length of ownership <2 months. The final sample included 427 relinquished dogs and 427 pet dogs that were successfully matched for sex, breed, and source of acquisition (in most cases). There were significant differences in neuter status (*X*^2^ = 5.53, *p* = 0.02) and age (*X*^2^ = 10.82, *p* = 0.01) between the groups, although *post-hoc* analyses based on standardized residuals were not statistically significant. Pet dog owners had owned their dogs for significantly longer at the time of completing the C-BARQ compared with relinquishing owners (*U* = 59,274.50, *Z* = −4.85, *p* < 0.001, Table 1).

**Table 1:** Characteristics of sample.

	**Relinquished dogs % (*n*)**	**Pet dogs % (*n*)**
**Sex**		
Male	55.5 (237)	55.5 (237)
Female	44.5 (190)	44.5 (190)
**Neuter status**		
Neutered	57.7 (246)	65.6 (280)
Entire	42.3 (180)	34.4 (147)
**Source of acquisition**		
Bred by owner	1.6 (7)	2.6 (11)
Breeder	22.2 (95)	20.8 (89)
Neighbor, friend, or relative	31.9 (136)	32.3 (138)
Other	12.4 (53)	13.3 (57)
Pet store	4.0 (17)	4.2 (18)
Shelter	21.1 (90)	19.7 (84)
Stray	6.8 (29)	7.0 (30)
**Age at evaluation**		
<6 months	4.2 (18)	4.4 (19)
6 months– <2 years	41.9 (179)	34.2 (146)
2 years– <8 years	44.7 (191)	45.7 (195)
>8 years	9.1 (39)	15.7 (67)
**Length of ownership** ^a^	18.00 (2.00–154.62)	31.99 (2.07–178.59)

a *Length of ownership was calculated based on the number of months between the dog's age at evaluation and their acquisition age. Data are shown as median (range)*.

**Figure 1:**
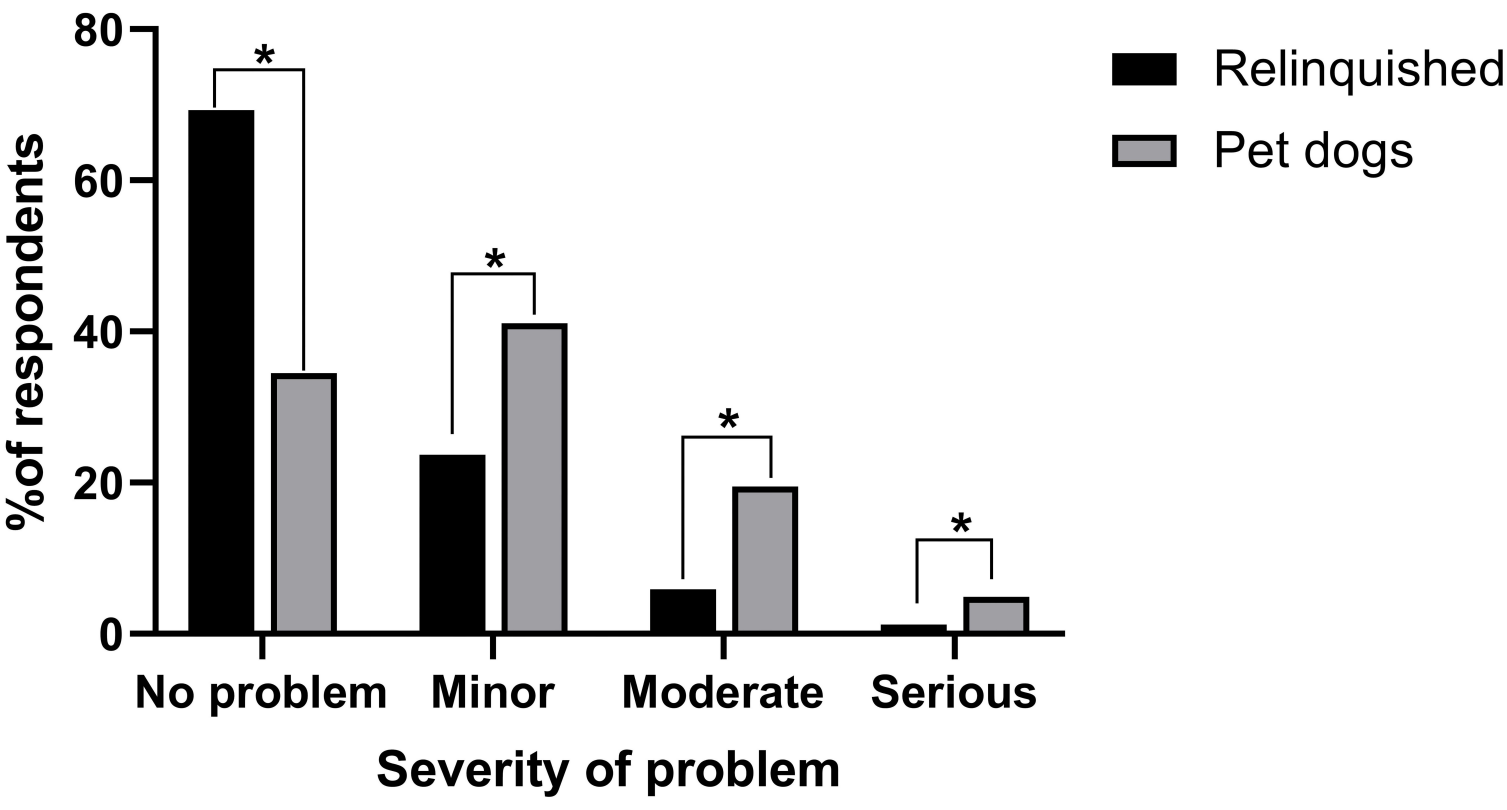
Relinquishing and pet dog owners' perceptions of dog behavior. ^*^ indicates statistical significance (*p* < 0.05).

**Figure 2:**
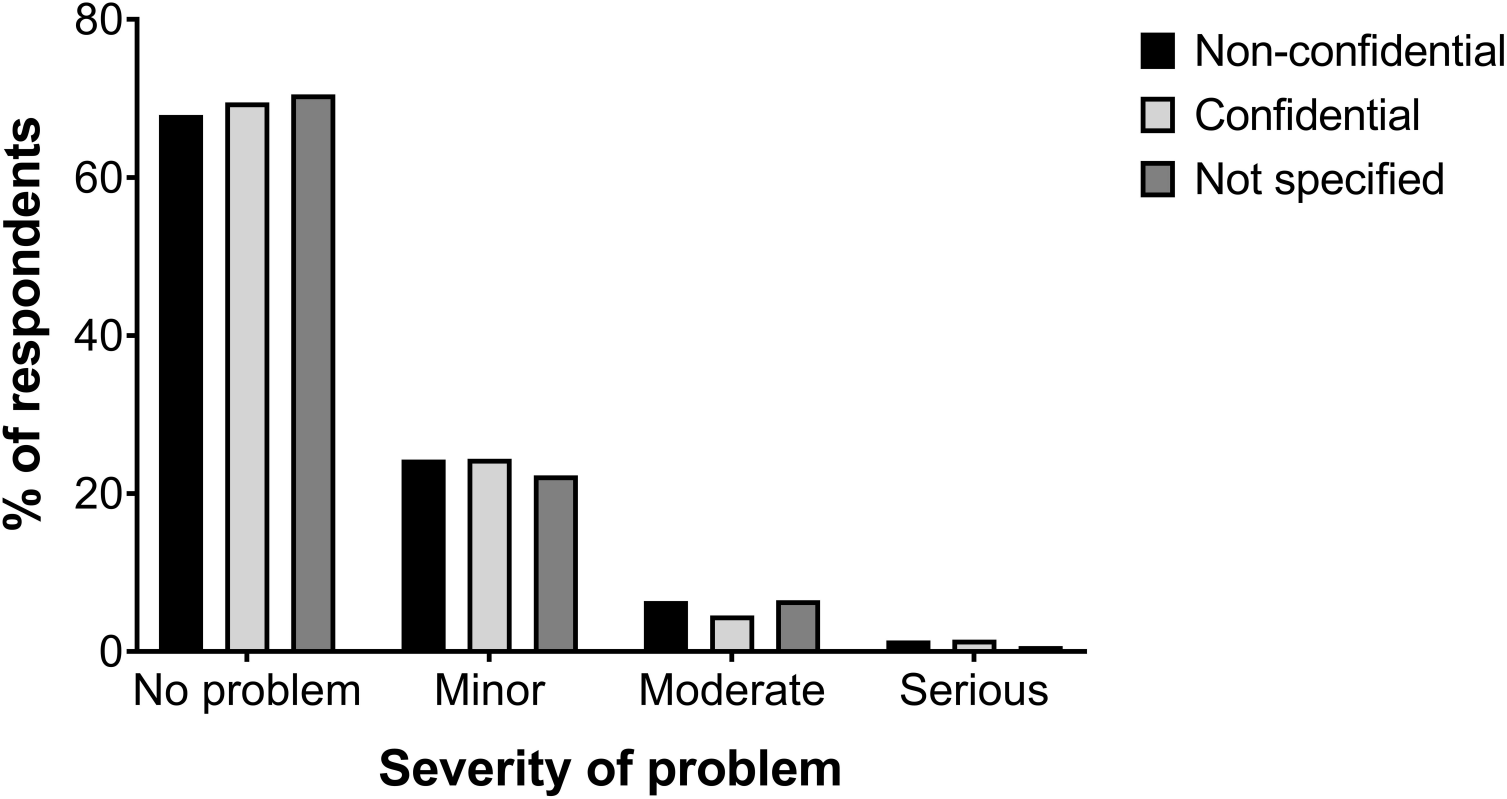
Relinquishing owners' perceptions of dog behavior based on the confidentiality of their survey responses.

Relinquishing owners and pet owners differed in their perceptions of their dogs' behavior (*X*^2^ = 108.03, *p* < 0.001, Figure 1). More than two-thirds of relinquishing owners reported their dog had no behavior problems (69.3%), 23.7% reported their dog had minor behavior problems, 5.9% reported moderate problems and 1.2% reported serious problems. Comparatively, 34.5% of pet owners reported their dogs had no behavior problems, 41.1% reported minor problems, 19.5% reported moderate problems and 4.9% reported serious problems. There were no differences in relinquishing owner's perceptions of their dog's behavior based on the confidentiality of their responses (*X*^2^ = 1.44, *p* = 0.97, Figure 2) or the shelter at which they relinquished their dog (*X*^2^ = 9.17, *p* = 0.14). The perceived confidentiality of relinquishing owners' responses was not associated with their dogs' mini C-BARQ subscale scores or miscellaneous items as described by Duffy et al. (20).

**Figure 3:**
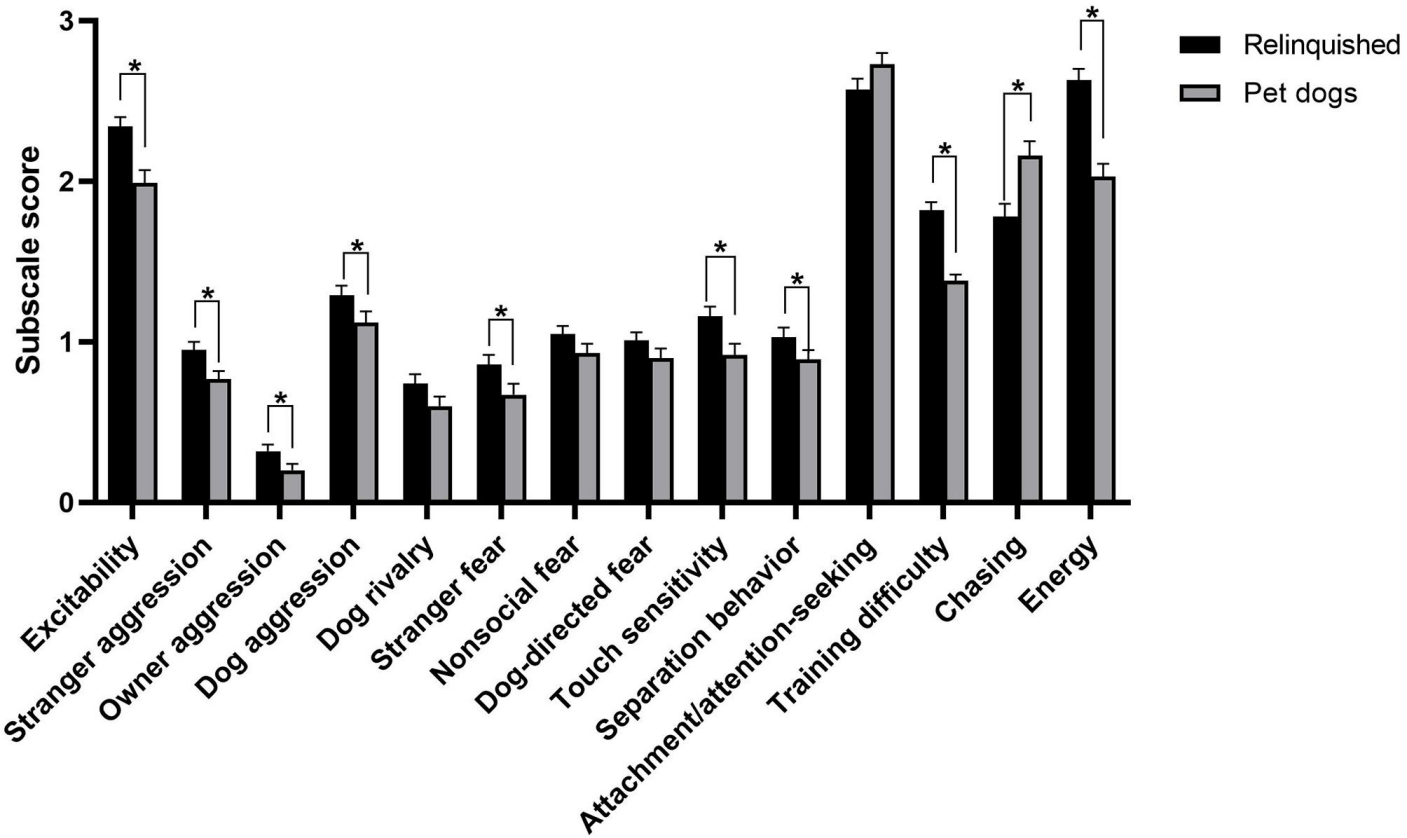
C-BARQ subscale scores between relinquished and pet dogs. Data are shown as mean ± SEM. ^*^ indicates statistical significance (*p* < 0.05).

**Figure 4:**
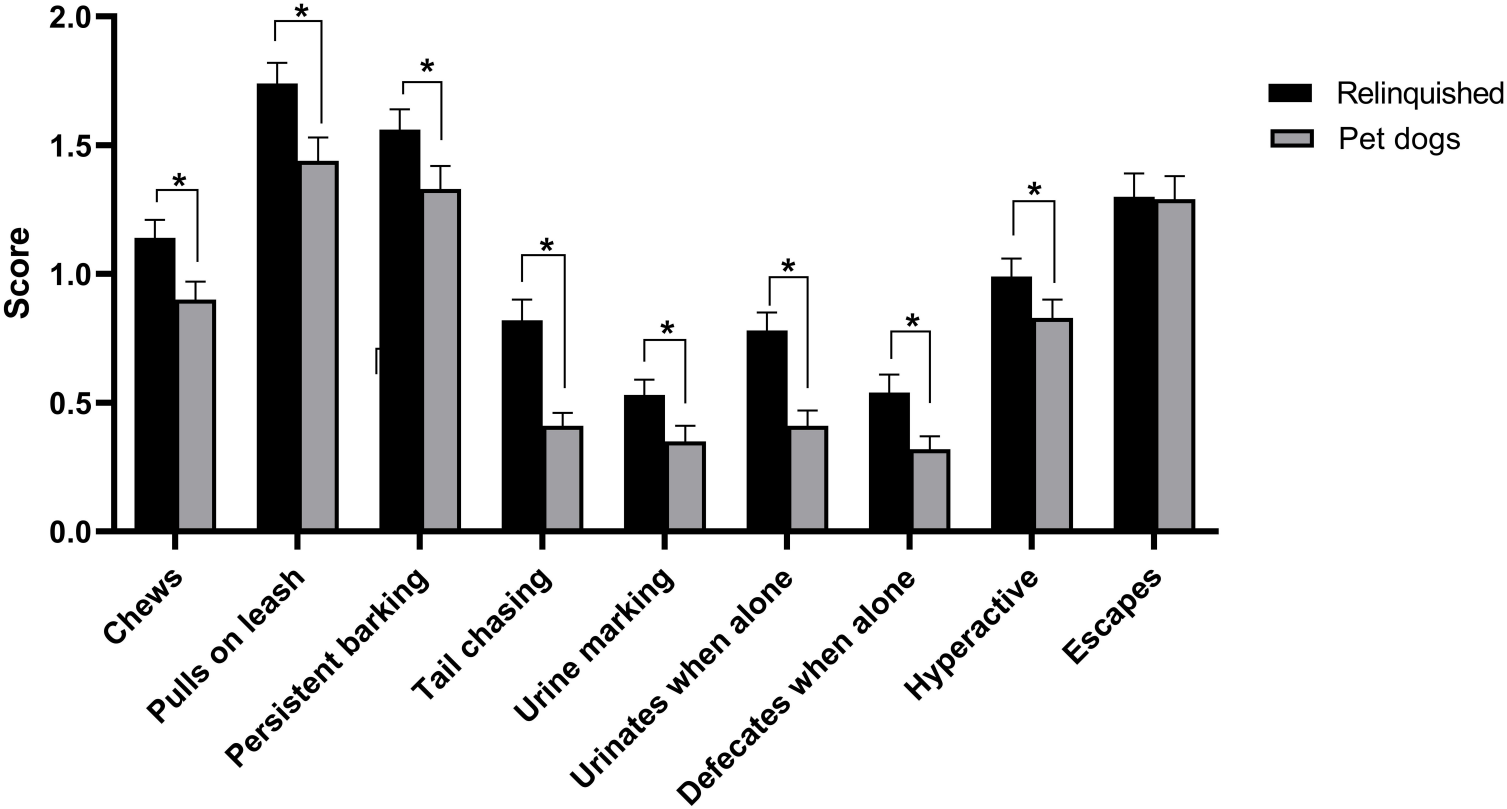
C-BARQ miscellaneous questions between relinquished and pet dogs. Data are shown as mean ± SEM. ^*^ indicates statistical significance (*p* < 0.05).

Relinquished dogs scored significantly higher than pet dogs on most C-BARQ subscales, including excitability [*t*_(830)_ = −5.76, *p* < 0.001], energy [*t*_(847)_ = −7.87, *p* < 0.001], training difficulty [*t*_(824)_ = −11.16, *p* < 0.001], stranger-directed aggression (*U* = 96,870, *z* = −3.92, and *p* < 0.001), owner-directed aggression (*U* = 100,314.50, *z* = −4.43, and *p* < 0.001), dog-directed aggression (*U* = 85,436.50, *z* = −2.69, and *p* = 0.01), stranger-directed fear (*U* = 94,441, *z* = −3.37, and *p* < 0.001), touch sensitivity (*U* = 69,390.50, *z* = −3.33, and *p* < 0.001), and separation-related behavior (*U* = 93,334, *z* = −2.04, and *p* = 0.04; Figure 3). There were no significant differences between relinquished and pet dogs in dog rivalry (*U* = 58,034, *z* = −1.36, and *p* = 0.18), dog-directed fear (*U* = 82,682, *z* = −1.83, and *p* = 0.07), nonsocial fear (*U* = 89,589, *z* = −1.78, and *p* = 0.07) and attachment/attention-seeking behavior (*U* = 82,186.50, *z* = −1.79, and *p* = 0.07), while pet dogs scored higher than relinquished dogs for chasing [*t*_(799)_ = 3.94, *p* < 0.001]. Relinquished dogs had significantly higher scores for all miscellaneous C-BARQ items, except for escaping behavior (*p* = 0.83; Figure 4).

The binary logistic regression models, including adjustment for sex, neuter status, age group and length of ownership, produced largely similar results (Table 2). Relinquished dogs scored significantly higher than pet dogs in excitability, stranger-directed aggression, owner-directed aggression, dog-directed aggression, touch sensitivity, training difficulty, energy, pulls on leash, tail chasing, urine marking, urinates when alone, and defecates when alone. Again, pet dogs had a significantly higher score than relinquished dogs on the chasing subscale, although they also scored higher for attachment/attention-seeking behavior.

**Table 2:** Association between relinquishment status and C-BARQ subscale or miscellaneous item scores.

**C-BARQ subscale**	***n* pet dogs**	***n* relinquished dogs**	**Adjusted odds ratio**	**95% confidence intervals**	***p*-value**
Excitability	416	339	1.54	1.33–1.77	<0.001^*^
Stranger-directed aggression	339	342	1.40	1.16–1.70	<0.001^*^
Owner-directed aggression	417	343	1.38	1.07–1.78	0.01^*^
Dog-directed aggression	385	327	1.19	1.03–1.38	0.02^*^
Dog rivalry (familiar dog aggression)	340	268	1.03	0.86–1.23	0.75
Stranger-directed fear	410	333	1.12	0.95–1.30	0.18
Nonsocial fear	402	341	1.05	0.88–1.25	0.61
Dog-directed fear	380	331	1.05	0.89–1.24	0.54
Touch sensitivity	357	282	1.24	1.06–1.45	0.01^*^
Separation-related behavior	417	337	1.06	0.90–1.24	0.51
Attachment/attention-seeking	423	340	0.86	0.74–1.00	0.05^*^
Training difficulty	406	343	3.34	2.56–4.36	<0.001^*^
Chasing	391	334	0.79	0.71–0.89	<0.001^*^
Energy	426	344	1.52	1.30–1.77	<0.001^*^
**Miscellaneous items**					
Chews	425	342	0.91	0.87–1.13	0.99
Pulls on leash	424	337	1.21	1.07–1.36	0.002^*^
Persistent barking	421	344	1.06	0.94–1.18	0.35
Tail chasing	423	342	1.20	1.04–1.38	0.01^*^
Urine marking	423	343	1.29	1.09–1.53	0.003^*^
Urinates when alone	419	336	1.24	1.07–1.43	0.004^*^
Defecates when alone	419	333	1.18	1.00–1.39	0.05^*^
Hyperactive	424	343	1.07	0.93–1.22	0.37
Escapes	399	342	0.95	0.85–1.06	0.33

*Logistic regression models were adjusted for sex, neuter status, age group, and length of ownership. Pet dogs were considered the referent category. Adjusted odds ratio show the odds of relinquishment based on a one-point increase in the C-BARQ subscale score or miscellaneous item*.

* *indicates there was a statistically significant difference (p < 0.05)*.

## Discussion

The goal of this study was to investigate whether relinquishing owners' reports of their dogs' behavior were influenced by the manner in which the topic was addressed at the time of relinquishment. We also examined how the perceived confidentiality of the information provided by relinquishing owners influenced their responses to different behavioral screening tools. Finally, we used a matching sample of currently owned dogs to determine if relinquishing and current (non-relinquishing) owners perceive the behavior of their dogs differently.

The results suggest that the perceived confidentiality of the behavioral information provided by relinquishing owners did not affect their responses to either the direct question regarding the dogs' behavior problems or the mini C-BARQ (20). Owners who believed their responses would be shared with the shelter staff and used for adoption decision-making were just as likely to report behavioral problems as owners who believed their data were confidential and would not be shared with the shelter. In contrast to the C-BARQ, which is designed to reduce subjective biases by asking owners to report the frequency or severity of behaviors in specific situations (26), the direct question might offer relinquishing owners greater opportunity to misrepresent their pet's behavior. However, as the perceived confidentiality did not impact relinquishing owners' responses to the direct question, our findings challenge the notion that relinquishing owners deliberately deceive shelter staff or provide biased information at the time of relinquishment.

At the same time, highly significant differences emerged in how relinquishing and current dog owners responded to the two types of behavioral assessments. Irrespective of the perceived confidentiality of their reports, relinquishing owners were far less likely to report experiencing minor, moderate, or serious behavior problems when asked directly compared with a matched sample of current dog owners. Conversely, the mini C-BARQ scores of currently owned dogs were significantly and consistently lower (more desirable) for the majority of subscales and miscellaneous items compared with those of the relinquished dogs. In other words, based on their mini C-BARQ scores, relinquished dogs had significantly less desirable behavior than the matching sample of currently owned dogs, but their owners were less likely to report behavioral issues when asked directly than pet owners, despite being unaffected by the perceived confidentiality of their responses.

If relinquishing owners were deliberately under-reporting their dog's behavior problems, we would anticipate the perceived confidentiality of their responses would impact the owner's reports, particularly to the direct question. However, this was not the case. The discrepancy between owners' responses to the direct question and the mini C-BARQ (where they had to describe specific behavioral responses to stimuli) could therefore indicate that relinquishing owners' were “honest” in their reporting or that they under-reported their dog's behavior irrespective of confidentiality because, (a) they failed to read or comprehend the confidentiality statements, or (b) they did not trust the stated claim that their responses would not be shared with the shelter.

If relinquishing owners were indeed reporting “honestly,” our findings may indicate that relinquishing owners were less informed about normal canine behavior and therefore did not always recognize their dog's behavior as a behavioral problem. Thus, when asked to describe specific behaviors using the mini C-BARQ, relinquishing owners reported behaviors that produced less desirable scores for their dogs than current pet owners. But, when asked directly about their experience of undesirable behavior, a general lack of understanding of what constitutes normal dog behavior could have meant they were less likely to report behavior problems than the sample of dog owners who voluntarily completed the C-BARQ. It is conceivable that relinquishing owners with a limited understanding of canine behavior were less likely to access behavioral resources due to a perceived lack of need. Previous research has found owners who do not attend obedience classes are at greater risk of relinquishing their pets (14, 16). Owners who do not attend puppy training classes are also less likely to report behaviors, such as pulling on a leash or separation-related behavior, as a problem (34).

It is also possible that some relinquishing owners faced difficulties accessing behavioral resources which may have hindered their understanding of canine behavior. Equity of access is a significant issue in veterinary medicine (35), and barriers, such as cost, transportation, and opening hours, can also affect pet owners' ability to access behavioral resources and knowledge (36). A recent study in the U.K. investigated the utilization of free training classes by low socioeconomic dog owners and found dog owners with lower household incomes had higher drop-out rates notwithstanding the removal of the cost barrier (36). The authors suggested that non-financial barriers likely prevented these underserved dog owners from attending training courses. Interventions that aim to increase access to behavioral resources must go beyond simply offering free services to combat other issues of inequality, such as transportation, job flexibility and timing of classes. Owners' demographic characteristics can also affect their perceptions of canine behaviors. Lord et al. (34) found owners who were employed or students were less likely to recognize canine behavioral problems than retired and unemployed owners. Demographic characteristics were not included in the present study to reduce the response burden on participants, but the possible influence of demographic characteristics on relinquishing owners' perceptions of dog behavior presents an interesting avenue for future research. The risk of relinquishment may also be affected by owner characteristics, such as gender and age (37, 38).

The possibility that relinquishing owners are unable to recognize canine behavior problems also presents an opportunity for animal shelters to develop intake diversion programs. Many shelters implement proactive relinquishment reduction programs to help pet owners who are considering relinquishment to retain their pets, thereby reducing the number of animals entering shelters. Community support programs (i.e., programs that aim to support general pet owners, including those who are not considering relinquishment) are also common among shelters. Examples include food banks and free/low-cost spay and neuter services (39–42). Our data suggest behavioral intake diversion programs might help to increase retention rates by assisting at-risk owners to recognize and manage their dog's behavioral problems, and could provide a crucial resource to improve equity of access for some dog owners. Behavioral intake diversion programs can take many forms from phone counselling to basic obedience training classes and one-on-one behavioral consultations. One example is the Richmond SPCA's behavioral helpline (41, 43). Community dog owners can call or email the helpline to receive behavioral support, enroll in behavioral classes and/or organize a face-to-face behavioral consultation. Although the program has not been formally evaluated, shelter staff report the helpline is heavily used (43).

In a recent survey of 111 animal shelters regarding community support and intake diversion programs, behavior training classes were listed as the top-ranking program that shelters believed would most help dog owners to retain their dogs, followed by behavior consultations (42). Only 27% of shelters in the study offered training classes and many indicated that financial and staffing restraints prevented them from implementing such programs (42). Research into relinquishment prevention programs is limited and studies focusing on behavioral diversion programs targeting at-risk dog owners are almost non-existent (44, 45). Considering the potentially costly nature of behavioral support programs, the need for animal shelters to efficiently utilize their resources and the considerable benefits that may be attained, the effectiveness of behavioral intake diversion programs should be considered a priority for future research.

Regardless of the underlying reasons for the discrepancy between relinquishing owners' responses to the mini C-BARQ and the direct question about their experience of behavior problems, the current findings indicate that the mini C-BARQ, or similar behavioral assessment tools, are likely to provide animal shelters with reliable and accurate information about a relinquished dog's behavior. The results of numerous previous studies suggest that behavior problems are a major reason why owners surrender or return dogs to shelters (12, 14–16). Our finding that relinquished dogs' mini C-BARQ scores were consistently poorer than those of a matched sample of pet dogs also suggests that behavioral issues might play a major role in relinquishment decisions.

This study is subject to several limitations that should be noted. Given the difference in recruitment strategies, the two groups of dog owners are likely to reflect different subsections of the dog owning population. Pet dog owners in this study were self-selected, meaning they voluntarily sought out and completed the C-BARQ online and likely had some level of interest in animal behavior. Relinquishing owners, on the other hand, were recruited to participate by shelter staff and did not necessarily have the same level of interest in understanding the behavior of their animals. The voluntary nature of the study meant some relinquishing owners may have declined to participate and the sample may not be representative of all relinquishing owners. As participants were recruited by animal shelter staff, we could not ascertain a response rate for this group to explore this issue. Similarly, the group of pet dog owners in this study may be subject to self-selection bias and may not be representative of the average dog owner given their aforementioned interest in animal behavior. Relinquishing and continuing pet dog owners also differed in terms of how they completed the mini C-BARQ (pencil-paper at the shelter compared with online) which may have impacted their responses. The groups of dogs differed in neuter status, age and length of ownership which could have affected their behavioral scores, although the binary logistic regression models including adjustment for these covariates produced similar results to the unadjusted analyses. The logistic regression models were subject to some missing values, particularly regarding the length of ownership, which resulted in the exclusion of some cases. Owners' responses to the direct question may have been impacted by order effects. All respondents answered the direct question prior to completing the mini C-BARQ/C-BARQ and it is possible that some owners would have answered the direct question differently had they completed the C-BARQ first. Finally, the reasons for relinquishment were not available to researchers which precluded an investigation of the level of agreement between owner's perceptions of dog behavior, their dog's C-BARQ scores and their relinquishment reasons. Interestingly, the 30% of relinquishing owners who reported canine behavioral problems mirrors several previous studies that found behavior problems were the primary relinquishment reason for ~30% of relinquishments (12, 13, 46). Nevertheless, relinquishment reasons are often multi-factorial (47) and future investigations of the associations between relinquishment reasons and C-BARQ scores could provide more information about relinquishing owners' perceptions of problem behavior.

## Conclusions

The findings of this study suggest that relinquishing owners provide more accurate information about their dog's behavior when asked about particular behaviors in specific circumstances and provide support for the use of validated behavioral questionnaires, such as the mini C-BARQ, to collect behavioral data from relinquishing owners at the time of relinquishment. When considering the owner's perceptions of their dog's behavior through direct question, we found most relinquishing owners reported they were not experiencing problems with their dog's behavior. The comparative sample of pet dog owners, on the other hand, were more likely to indicate their dogs were exhibiting mild, moderate or serious behavior problems. In contrast, relinquishing owners' responses to the mini C-BARQ indicated their dogs were exhibiting higher rates of undesirable behavior compared with the matched sample of pet dogs. The disparity between relinquishing owners' reports of their dog's behavior based on the direct question and the mini C-BARQ does not appear to be attributable to deliberately biased reporting from relinquishing owners as the perceived confidentiality (or lack thereof) did not affect relinquishing owner's responses regarding their dog's behavior. It is therefore possible that relinquishing owners had less understanding of dog behavior and did not recognize their dog's behaviors as problem behaviors. Animal shelters could increase owners' retention of their animals by implementing behavioral intake diversion programs to support dog owners who are at risk of relinquishment and help them to manage their pets' behavior.

## Data Availability Statement

The data analyzed in this study is subject to the following licenses/restrictions: The data are freely available on request to the corresponding author. Requests to access these datasets should be directed to serpell@vet.upenn.edu.

## Ethics Statement

The studies involving human participants were reviewed and approved by University of Pennsylvania Institutional Review Board. Written informed consent for participation was not required for this study in accordance with the national legislation and the institutional requirements.

## Author Contributions

JS conceived and designed the study. KK collected and compiled the data. DD performed preliminary analyses. LP conducted the final statistical analyses and drafted the manuscript. JS, LP, and BW interpreted the data. All authors contributed to manuscript revision, read, and approved the submitted version.

## Funding

This research was supported by a Morris Animal Foundation grant #D07CA-071 to JS. LP's position is supported by the Arnall Family Foundation.

## Conflict of Interest

The authors declare that the research was conducted in the absence of any commercial or financial relationships that could be construed as a potential conflict of interest.

## Publisher's Note

All claims expressed in this article are solely those of the authors and do not necessarily represent those of their affiliated organizations, or those of the publisher, the editors and the reviewers. Any product that may be evaluated in this article, or claim that may be made by its manufacturer, is not guaranteed or endorsed by the publisher.

## References

[1] Dinwoodie IR Dwyer B Zottola V Gleason D Dodman NH. Demographics and comorbidity of behavior problems in dogs. J Vet Behav. (2019) 32:62–71. 10.1016/j.jveb.2019.04.007

[2] Salonen M Sulkama S Mikkola S Puurunen J Hakanen E Tiira K. Prevalence, comorbidity, and breed differences in canine anxiety in 13,700 Finnish pet dogs. Sci Rep. (2020) 10:1–11. 10.1038/s41598-020-59837-z 32139728 PMC7058607

[3] Landsberg G Hunthausen W Ackerman L. Behavior Problems of the Dog and Cat-E-Book. Edinburgh; London; New York, NY; Oxford; Philadelphia, PA; St Louis, MO; Sydney, NSW; Toronto, ON: Elsevier Health Sciences (2011).

[4] Stephen JM Ledger RA. An audit of behavioral indicators of poor welfare in kenneled dogs in the United Kingdom. J Appl Anim Welf Sci. (2005) 8:79–95. 10.1207/s15327604jaws0802_1 16277592

[5] Beerda B Schilder MB van Hooff JA de Vries HW. Manifestations of chronic and acute stress in dogs. Appl Anim Behav Sci. (1997) 52:307–19. 10.1016/S0168-1591(96)01131-8

[6] Boyd C Jarvis S McGreevy P Heath S Church D Brodbelt D. Mortality resulting from undesirable behaviours in dogs aged under three years attending primary-care veterinary practices in England. Anim Welf. (2018) 27:251–62. 10.7120/09627286.27.3.251

[7] Yu Y Wilson B Masters S van Rooy D McGreevy PD. Mortality resulting from undesirable behaviours in dogs aged three years and under attending primary-care veterinary practices in Australia. Animals. (2021) 11:493. 10.3390/ani11020493 33668532 PMC7918417

[8] Kwan JY Bain MJ. Owner attachment and problem behaviors related to relinquishment and training techniques of dogs. J Appl Anim Welf Sci. (2013) 16:168–183. 10.1080/10888705.2013.768923 23544756

[9] González-Ramírez MT Vanegas-Farfano M Landero-Hernández R. Differences in stress and happiness between owners who perceive their dogs as well behaved or poorly behaved when they are left alone. J Vet Behav. (2018) 28:1–5. 10.1016/j.jveb.2018.07.010

[10] Herwijnen IRv van der Borg JA Naguib M Beerda B. Dog ownership satisfaction determinants in the owner-dog relationship and the dog's behaviour. PLoS One. (2018) 13:e0204592. 10.1371/journal.pone.0204592 30235347 PMC6147508

[11] Serpell JA. Evidence for an association between pet behavior and owner attachment levels. Appl Anim Behav Sci. (1996) 47:49–60. 10.1016/0168-1591(95)01010-6

[12] Diesel G Brodbelt D Pfeiffer DU. Characteristics of relinquished dogs and their owners at 14 rehoming centers in the United Kingdom. J Appl Anim Welf Sci. (2010) 13:15–30. 10.1080/10888700903369255 20017043

[13] Salman M New J John G Scarlett JM Kass PH Ruch-Gallie R. Human and animal factors related to relinquishment of dogs and cats in 12 selected animal shelters in the United States. J Appl Anim Welf Sci. (1998) 1:207–26. 10.1207/s15327604jaws0103_2 16363966

[14] Patronek GJ Glickman LT Beck AM McCabe GP Ecker C. Risk factors for relinquishment of dogs to an animal shelter. J Am Vet Med Assoc. (1996) 209:572–81. 8755975

[15] Powell L Reinhard C Satriale D Morris M Serpell J Watson B. Characterizing unsuccessful animal adoptions: age and breed predict the likelihood of return, reasons for return and post-return outcomes. Sci Rep. (2021) 11:8018. 10.1038/s41598-021-87649-2 33850258 PMC8044234

[16] Diesel G Pfeiffer D Brodbelt D. Factors affecting the success of rehoming dogs in the UK during 2005. Prev Vet Med. (2008) 84:228–41. 10.1016/j.prevetmed.2007.12.004 18243374

[17] Wells DL Hepper PG. Prevalence of behaviour problems reported by owners of dogs purchased from an animal rescue shelter. Appl Anim Behav Sci. (2000) 69:55–65. 10.1016/S0168-1591(00)00118-0 10856784

[18] Clay L Paterson M Bennett P Perry G Rohlf V Phillips CJ. In defense of canine behavioral assessments in shelters: outlining their positive applications. J Vet Behav. (2020) 38:74–81. 10.1016/j.jveb.2020.03.005

[19] Patronek GJ Bradley J. No better than flipping a coin: reconsidering canine behavior evaluations in animal shelters. J Vet Behav. (2016) 15:66–77. 10.1016/j.jveb.2016.08.001

[20] Duffy DL Kruger KA Serpell JA. Evaluation of a behavioral assessment tool for dogs relinquished to shelters. Prev Vet Med. (2014) 117:601–9. 10.1016/j.prevetmed.2014.10.003 25457136

[21] Segurson SA Serpell JA Hart BL. Evaluation of a behavioral assessment questionnaire for use in the characterization of behavioral problems of dogs relinquished to animal shelters. J Am Vet Med Assoc. (2005) 227:1755–61. 10.2460/javma.2005.227.1755 16342523

[22] Clay L Paterson M Bennett P Perry G Phillips CC. Comparison of canine behaviour scored using a shelter behaviour assessment and an owner completed questionnaire, C-BARQ. Animals. (2020) 10:1797. 10.3390/ani10101797 33022960 PMC7600298

[23] Bennett SL Litster A Weng H-Y Walker SL Luescher AU. Investigating behavior assessment instruments to predict aggression in dogs. Appl Anim Behav Sci. (2012) 141:139–48. 10.1016/j.applanim.2012.08.005

[24] DiGiacomo N Arluke A Patronek G. Surrendering pets to shelters: the relinquisher's perspective. Anthrozoös. (1998) 11:41–51. 10.1080/08927936.1998.11425086

[25] Stephen J Ledger R. Relinquishing dog owners' ability to predict behavioural problems in shelter dogs post adoption. Appl Anim Behav Sci. (2007) 107:88–99. 10.1016/j.applanim.2006.09.012

[26] Hsu Y Serpell JA. Development and validation of a questionnaire for measuring behavior and temperament traits in pet dogs. J Am Vet Med Assoc. (2003) 223:1293–300. 10.2460/javma.2003.223.1293 14621216

[27] Mariti C Gazzano A Moore JL Baragli P Chelli L Sighieri C. Perception of dogs' stress by their owners. J Vet Behav. (2012) 7:213–9. 10.1016/j.jveb.2011.09.004

[28] Wan M Bolger N Champagne FA. Human perception of fear in dogs varies according to experience with dogs. PLoS One. (2012) 7:e51775. 10.1371/journal.pone.0051775 23284765 PMC3526646

[29] Tami G Gallagher A. Description of the behaviour of domestic dog (*Canis familiaris*) by experienced and inexperienced people. Appl Anim Behav Sci. (2009) 120:159–169. 10.1016/j.applanim.2009.06.009

[30] Powell L Stefanovski D Siracusa C Serpell J. Owner personality, owner-dog attachment, and canine demographics influence treatment outcomes in canine behavioral medicine cases. Front Vet Sci. (2021) 7:1238. 10.3389/fvets.2020.630931 33553291 PMC7862121

[31] Duffy DL Serpell JA. Predictive validity of a method for evaluating temperament in young guide and service dogs. Appl Anim Behav Sci. (2012) 138:99–109. 10.1016/j.applanim.2012.02.011

[32] McGreevy PD Georgevsky D Carrasco J Valenzuela M Duffy DL Serpell JA. Dog behavior co-varies with height, bodyweight and skull shape. PLoS One. (2013) 8:e80529. 10.1371/journal.pone.0080529 24358107 PMC3864788

[33] Dodman NH Brown DC Serpell JA. Associations between owner personality and psychological status and the prevalence of canine behavior problems. PLoS One. (2018) 13:e192846. 10.1371/journal.pone.0192846 29444154 PMC5812720

[34] Lord MS Casey RA Kinsman RH Tasker S Knowles TG Da Costa RE. Owner perception of problem behaviours in dogs aged 6 and 9-months. Appl Anim Behav Sci. (2020) 232:105147. 10.1016/j.applanim.2020.105147

[35] LaVallee E Mueller MK McCobb E. A systematic review of the literature addressing veterinary care for underserved communities. J Appl Anim Welf Sci. (2017) 20:381–94. 10.1080/10888705.2017.1337515 28657796

[36] Harris L Durston T Flatman J Kelly D Moat M Mohammed R. Impact of socio-economic status on accessibility of dog training classes. Animals. (2019) 9:849. 10.3390/ani9100849 31652507 PMC6826670

[37] New JC Jr Salman M King M Scarlett JM Kass PH Hutchison JM. Characteristics of shelter-relinquished animals and their owners compared with animals and their owners in US pet-owning households. J Appl Anim Welf Sci. (2000) 3:179–201. 10.1207/S15327604JAWS0303_1

[38] Dolan ED Scotto J Slater M Weiss E. Risk factors for dog relinquishment to a Los Angeles municipal animal shelter. Animals. (2015) 5:1311–28. 10.3390/ani5040413 26690483 PMC4693217

[39] Humane Society of the United States. Pets for Life - A New Community Understanding. (2012). Available online at: https://www.humanesociety.org/sites/default/files/docs/2012-pets-for-life-report.pdf (cited March 4, 2021).

[40] ASPCA. Safety Net Programs. (2021). Available online at: https://www.aspcapro.org/resource/safety-net-programs (cited April 20, 2021).

[41] Weiss E. Safety nets and support for pets at risk of entering the sheltering system. Anim Behav Shelter Vet Staff . (2015) 286–91. 10.1002/9781119421313.ch15

[42] Russo A Dowling-Guyer S McCobb E. Community programming for companion dog retention: a survey of Animal Welfare Organizations. J Appl Anim Welf Sci. (2021). 10.1080/10888705.2020.1869551. [Epub ahead of print]. 33530720

[43] ASPCA. Richmond SPCA: Behavior Helpline & Behavior Classes. (2007). Available online at: https://www.aspcapro.org/sites/default/files/rspca_behavior_helpline.pdf (cited April 21, 2021).

[44] Protopopova A Gunter L. Adoption and relinquishment interventions at the animal shelter: a review. Anim Welf. (2017) 26:35–48. 10.7120/09627286.26.1.035

[45] Coe JB Young I Lambert K Dysart L Nogueira Borden L Rajić A. A scoping review of published research on the relinquishment of companion animals. J Appl Anim Welf Sci. (2014) 17:253–73. 10.1080/10888705.2014.899910 24738944

[46] Miller DD Staats SR Partlo C Rada K. Factors associated with the decision to surrender a pet to an animal shelter. J Am Vet Med Assoc. (1996) 209:738–42. 8756871

[47] Weiss E Slater M Garrison L Drain N Dolan E Scarlett JM. Large dog relinquishment to two municipal facilities in New York City and Washington, DC: Identifying targets for intervention. Animals. (2014) 4:409–33. 10.3390/ani4030409 26480315 PMC4494313

