# Supplementary material for: Relinquishing Owners Underestimate Their Dog's Behavioral Problems: Deception or Lack of Knowledge?
Source: Front Vet Sci. 2021 Sep 10;8:734973. doi: 10.3389/fvets.2021.734973 (PMC8461173; doi:10.3389/fvets.2021.734973)
Supplement: Supplementary file 1 [file Table_1.DOCX]

Supplementary Material

**Supplementary Table 1.** Description of C-BARQ subscales and miscellaneous items.

| **C-BARQ scale** | **Questions per scale** | **Description** |
| --- | --- | --- |
| Excitability | 2 | Strong reactions to arousing events, e.g., being taken on a walk or on a car trip. |
| Stranger-directed aggression | 3 | Aggressive behavior/s towards unfamiliar people while in the home, outside/yard or on a leash. |
| Owner-directed aggression | 3 | Aggressive behaviors towards owner when approached while eating or in possession of other high value objects. |
| Dog-directed aggression | 2 | Aggressive behaviors toward unfamiliar dogs. |
| Dog rivalry (familiar dog aggression) | 2 | Aggressive behaviors towards household dogs. |
| Stranger-directed fear | 2 | Fearful behaviors when approached by unfamiliar people. |
| Nonsocial fear | 3 | Fearful behaviors in response to loud noises, unfamiliar objects, and unfamiliar situations. |
| Dog-directed fear | 2 | Fearful behaviors towards unfamiliar dogs. |
| Touch sensitivity | 2 | Fearful behaviors when having nails clipped, being groomed, or bathed by a household member. |
| Separation-related behavior | 3 | Signs of anxiety, vocalization or destructive behavior while separated from the owner. |
| Attachment/attention-seeking | 2 | Tendency to solicit attention and maintain close proximity to owner. |
| Training difficulty | 3 | Tendency to become easily distracted or ignore owner commands. |
| Chasing | 2 | Tendency to chase small animals, e.g., birds, squirrels, rabbits. |
| Energy | 2 | Playful, boisterous, active, and always on the go. |

**Supplementary Table 2.** Number of valid cases for each mini C-BARQ subscale or miscellaneous item.

|  | **Pet dogs** | **Relinquished dogs** |
| --- | --- | --- |
| **C-BARQ scale** |  |  |
| Excitability | 416 | 416 |
| Stranger-directed aggression | 399 | 420 |
| Owner-directed aggression | 417 | 421 |
| Dog-directed aggression | 385 | 400 |
| Dog rivalry (familiar dog aggression) | 340 | 323 |
| Stranger-directed fear | 410 | 408 |
| Nonsocial fear | 402 | 416 |
| Dog-directed fear | 380 | 405 |
| Touch sensitivity | 357 | 340 |
| Separation-related behavior | 417 | 414 |
| Attachment/attention-seeking | 423 | 418 |
| Training difficulty | 406 | 420 |
| Chasing | 391 | 410 |
| Energy | 426 | 423 |
| **C-BARQ miscellaneous items** |  |  |
| Chews inappropriate objects | 425 | 421 |
| Pulls on leash | 424 | 415 |
| Persistent barking | 421 | 422 |
| Tail chasing | 423 | 421 |
| Urine marking | 423 | 420 |
| Urinates when left alone | 419 | 410 |
| Defecates when alone | 419 | 406 |
| Hyperactive | 424 | 422 |
| Escapes from home/yard | 399 | 418 |

**Supplementary Table 3.** Dog breeds included in the study.

|  | **Pet dogs** | **Relinquished dogs** | **Total** |
| --- | --- | --- | --- |
| **Breed** |  |  |  |
| Airedale Terrier | 2 | 2 | 4 |
| Akita | 11 | 11 | 22 |
| Alaskan Malamute | 1 | 1 | 2 |
| American Bulldog | 5 | 5 | 10 |
| American Pit Bull Terrier | 14 | 14 | 28 |
| American Staffordshire Terrier | 1 | 1 | 2 |
| Australian Cattle Dog | 8 | 8 | 16 |
| Australian Shepherd | 7 | 7 | 14 |
| Basenji | 1 | 1 | 2 |
| Basset Hound | 1 | 1 | 2 |
| Beagle | 14 | 14 | 28 |
| Bearded Collie | 1 | 1 | 2 |
| Bichon Frise | 3 | 3 | 6 |
| Border Collie | 9 | 9 | 18 |
| Border Terrier | 1 | 1 | 2 |
| Boston Terrier | 3 | 3 | 6 |
| Boxer | 17 | 17 | 34 |
| Bull Terrier | 1 | 1 | 2 |
| Cairn Terrier | 1 | 1 | 2 |
| Cane Corso | 1 | 1 | 2 |
| Chihuahua | 16 | 16 | 32 |
| Chow Chow | 9 | 9 | 18 |
| Cocker Spaniel | 6 | 6 | 12 |
| Collie (Smooth) | 3 | 3 | 6 |
| Coonhound | 2 | 2 | 4 |
| Corgi | 3 | 3 | 6 |
| Curr | 2 | 2 | 4 |
| Dachshund | 3 | 3 | 6 |
| Doberman Pinscher | 1 | 1 | 2 |
| English Springer Spaniel | 1 | 1 | 2 |
| French Bulldog | 1 | 1 | 2 |
| German Shepherd | 32 | 32 | 64 |
| German Shorthaired Pointer | 1 | 1 | 2 |
| Golden Retriever | 6 | 6 | 12 |
| Great Dane | 2 | 2 | 4 |
| Greyhound | 1 | 1 | 2 |
| Havanese | 1 | 1 | 2 |
| Hound mix | 4 | 4 | 8 |
| Irish Setter | 1 | 1 | 2 |
| Italian Greyhound | 2 | 2 | 4 |
| Jack Russell Terrier | 6 | 6 | 12 |
| Keeshond | 2 | 2 | 4 |
| Labrador Retriever | 84 | 84 | 168 |
| Lhasa Apso | 5 | 5 | 10 |
| Manchester Terrier | 2 | 2 | 4 |
| Mastiff (English) | 2 | 2 | 4 |
| Miniature Pinscher | 3 | 3 | 6 |
| Mixed Breed/Unknown | 5 | 5 | 10 |
| Neapolitan Mastiff | 3 | 3 | 6 |
| Newfoundland | 1 | 1 | 2 |
| Parson Russell Terrier | 5 | 5 | 10 |
| Pharaoh Hound | 1 | 1 | 2 |
| Pit Bull | 37 | 37 | 74 |
| Pit Bull mix | 6 | 6 | 12 |
| Pointer | 2 | 2 | 4 |
| Pomeranian | 3 | 3 | 6 |
| Poodle | 10 | 10 | 20 |
| Pug | 6 | 6 | 12 |
| Rat Terrier | 1 | 1 | 2 |
| Rhodesian Ridgeback | 2 | 2 | 4 |
| Rottweiler | 10 | 10 | 20 |
| Saint Bernard | 1 | 1 | 2 |
| Schipperke | 1 | 1 | 2 |
| Schnauzer | 4 | 4 | 8 |
| Shar Pei | 2 | 2 | 4 |
| Shetland Sheepdog | 1 | 1 | 2 |
| Shih Tzu | 6 | 6 | 12 |
| Siberian Husky | 9 | 9 | 18 |
| Weimaraner | 2 | 2 | 4 |
| West Highland White Terrier | 2 | 2 | 4 |
| Whippet | 1 | 1 | 2 |
| Yorkshire Terrier | 3 | 3 | 6 |
| **Total** | **427** | **427** | **854** |
